# Ultrasonography of the neotropical primate female reproductive system

**DOI:** 10.3389/fvets.2023.1214509

**Published:** 2024-03-08

**Authors:** Sheyla Farhayldes Souza Domingues, Danuza Leite Leão, Frederico Ozanan Barros Monteiro

**Affiliations:** ^1^Laboratory of Wild Animal Biotechnology and Medicine, Veterinary Medicine Institute, Federal University of Pará, Castanhal, State of Pará, Brazil; ^2^Mamirauá Institute for Sustainable Development, Tefé, State of Amazonas, Brazil; ^3^Laboratory of Animal Physiology, Health and Animal Production Institute, Federal Rural University of the Amazon, Belém, State of Pará, Brazil

**Keywords:** ultrasonography, neotropical primates, reproduction, female, ovary, gestation, triplex Doppler

## Abstract

The Neotropical (e. g., *Aotus* sp., *Callithrix jacchus, Saguinus* sp., *Saimiri* sp., and *Sapajus* sp.) primates are important models for biomedical research and studies on reproductive physiology and biotechnology. Consequently, studies about gynecological and obstetric ultrasonography are crucial. B-mode ultrasonography is a non-invasive imaging technique that provides real-time bidimensional or three-dimensional/four-dimensional B-mode images. In association with Doppler ultrasonography, B-mode ultrasonography can also be used to monitor the mammalian blood flow to the reproductive tract during important events such as ovulation and gestation. Thus, gynecological and obstetric ultrasonography is essential for establishing the female reproductive anatomical and physiological ovarian and uterine health status, gestational diagnosis, and fetal growth monitoring. For instance, the paper presents and discusses the state-of-the-art gynecological and obstetric ultrasonography in the Neotropical primates, species that are models for biomedical research, and some recent studies on species targets for conservation strategies for wild animal populations.

## 1 Introduction

**Figure 1:**
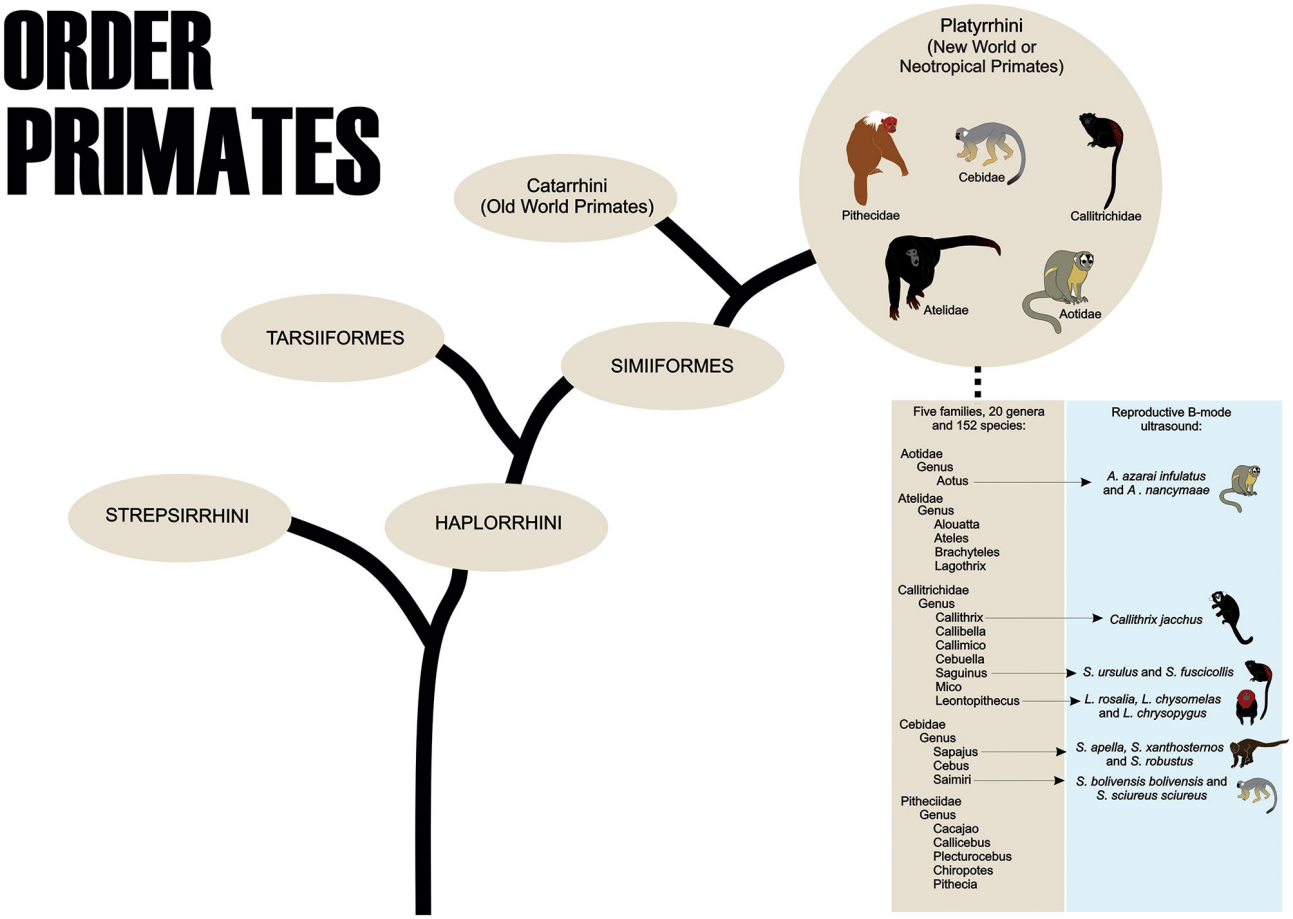
The order Primates shows the taxonomic classification of Neotropical Primates and the main species used in B-mode ultrasound studies of the female reproductive system.

Primates are part of the Order Primates, divided into two Suborders, Strepsirrhini and Haplorrhini. The Haplorrhini comprises the infraorders Tarsiiformes (tarsiers) and Simiiformes (apes, monkeys, and humans) (1). About the Simiiformes, these non-human primates are divided into two Parvorders, the Catarrhini, which comprises the species of monkeys known as Primates of the Old World that inhabit regions of Africa and Asia, and the Platyrrhini, which contains the species that inhabit areas of Mexico and the Americas Central and South that are called the New World or Neotropical Primates (2). The Neotropical primates are currently distributed among 152 species (204 species and subspecies), 20 genera, and five families: Cebidae, Aotidae, Pitheciidae, Atelidae, and Callitrichidae (Figure 1) (3).

To the best of our knowledge, in neotropical primates, the reproductive B-mode ultrasound examination has been described in the Aoetidae, in the genus Aotus (*Aotus azarai infulatus* and *A. nancymaae*), in the Callitrichidae, genus Callithrix (*Callithrix jacchus*), Saguinus (Saguinus *ursulus* and *S. fuscicollis*) and Leontopithecus (*Leontopithecus rosalia, L. chysomelas* and *L. chrysopygus*), and in the Cebidae, in subfamily Cebinae, genus Sapajus (*Sapajus apella, S. xanthosternos*, and *S. robustus*), and subfamily Saimiriinae, genus Saimiri (*Saimiri bolivensis bolivensis, S. sciureus sciureus*) as described in Table 1.

**Table 1:** Types of transducers and frequency (MHz) used for reproductive ultrasound examination in neotropical primate female according to species and animal body mass (kg).

**Species**	**Body mass (kg)**	**Transducer frequency (MHz)**	**Transducer types**	**References**
*Aotus azarai infulatus*	0.951 ± 0.096	7.5	Linear	(4)
	-	5–12	Linear	(5)
	-	6–18	Linear	(6)
	-	10–18	Linear	(7)
	-	7–12	Linear	(8)
*Aotus nancymaae*	-	5–13	Linear	(9)
*Callithrix jacchus*	-	7.5 or 10	Mechanical probes	(10)
	0.450	7.5 or 10	Mechanical probes	(11)
	0.351 ± 0.048	7.5	Linear	(12)
	-	7.5	Linear and convex sector probes	(13)
	-	10 and 7.5	Mechanical	(14)
	0.284–0.534		Linear	(15)
	0.350	3–9	Linear	(16)
	0.300–0.540	-	Linear	(17)
*Cebus albifrons*	2.75–3.50	7.5 and 4–9	Convexo and microconvexo multifrequencial	(18)
*Saguinus ursulus*	0.280–0.595	8–18	Linear	(19)
*Saguinus fuscicollis*		10–7.5	Sectorial	(20)
	0.140–0.800	7.5	-	(21)
*Saimiri bolivensis bolivensis*	-	6–15	Linear	(8)
	< 1	6–15	Linear	(8)
*Saimiri sciureus sciureus*	0.739	6.5		(22)
*Sapajus* sp. (*Cebus apella*)		5	Linear	(23)
	2–3	7.5	Convex	(24)
*Sapajus paella*	2.20	7–11	Microconvex	(10)
	-	5–8 and 5–12	Multifrequencial microconvex and linear	(25)
*Sapajus xanthosternos* and *Sapajus robustus*	-	5–8 and 5–12	Linear	(26)
*Leontopithecus* sp. (*L. rosalia*; *L. chysomelas; L. chrysopygus*)	-	7.5	Linear	(27)

Ultrasonography is a non-invasive imaging technique that provides real-time bidimensional (2D) or three-dimensional/four-dimensional (3D/4D) B-mode images (brightness) (28, 29); generates high-resolution characterization of reproductive organs; and provides precise linear measurements of the ovary, uterus, conceptus, and fetus (30). In association with Doppler ultrasonography, B-mode ultrasonography can also monitor mammalian blood flow to the reproductive tract (31, 32). Color ultrasonography and spectral Doppler ultrasonography can provide qualitative and quantitative data concerning blood flow, respectively (33). Color ultrasonography and spectral Doppler ultrasonography were used to detect hemodynamic changes in the iliac (6), uterine, ovary, and utero-ovarian ligament (UOL) arteries during follicular growth and ovulation, as well as the formation and regression of the corpus luteum (CL) (10, 25). In obstetrics, color ultrasonography and spectral Doppler ultrasonography are crucial for evaluating strategic conceptus vessels by fetal development (25). Nowadays, portable ultrasound equipment provides a vast possibility of modes (2D/3D/4D and color/spectral/power Doppler) and is reliable in animal research institutes, zoos, and field studies (34).

Gynecological and obstetric ultrasonography is essential for establishing the female reproductive anatomical and physiological ovarian and uterine health status (10), gestational diagnosis, and fetal growth monitoring (25). Ultrasound has become an indispensable method for routine management, health assessment, and research on the reproduction of non-human primates (NHPs) in captivity (35). Uterine and ovarian examinations have always been of great concern in gynecology because they can host numerous diseases related to reduced fertility or cause serious damage to the health of females (6, 36). Several techniques, such as ultrasonography, have been used to detect pregnancy in mammals, some of which represent technological advances in practice with captive animals (27). The increasing use of pregnant primates and their fetuses in scientific research makes diagnosing and monitoring pregnancy important, as it enables proper handling of the pregnant female (37). In the Neotropical primates, researchers have recently been developing gynecological (Tables 2, 3) and obstetric (Table 4) ultrasonography data to improve the reproductive management of these animals in captivity.

**Table 2:** Mean and standard deviation of the non-pregnant uterine measurements in neotropical adult primates by ultrasound examination.

**Species**	**Length (cm) (CCD)**	**Heigh (cm) (DVD)**	**Width (cm) (TD)**	**Uterus volume (cm^3^)**	**References**
*Aotus azarai infulatus*					(4)
Primiparous	1.85 ± 0.13	5.40 ± 0.59	9.10 ± 0.59	0.476 ± 0.0	
Multiparous	1.91 ± 0.14	5.73 ± 0.70	9.32 ± 0.58	0.540 ±0.10	
*Aotus azarai infulatus*					(5)
Nuliparous	1.74 ±0.11	0.50 ± 0.04	0.74 ± 0.05	0.33 ± 0.03	
Primiparous	1.73 ± 0.14	0.54 ± 0.04	0.78 ± 0.07	0.39 ± 0.07	
Multiparous	1.78 ± 0.21	0.67 ± 0.09	0.88 ± 0.07	0.56 ± 0.13	
*Sapajus apella* (*Cebu apella*)	1.79 ± 0.04	1.24 ± 0.03	1.36 ± 0.03	1.55 ± 0.08	(10)
Alfa S. xanthosternos/S. robustus^*^	1.74 ± 0.31	1.10 ± 0.17	1.05 ± 0.16	1.06 ± 0.43	(26)
Beta S. xanthosternos/S. robustus^**^	1.70 ± 0.52	1.01 ± 0.33	1.04 ± 0.27	0.97 ± 0.55	(26)
*Saguinus ursulus*	1.78 ± 0.34	0.46 ± 0.10	0.90 ± 0.36	0.41 ± 0.18	(19)
*Saguinus fuscicollis*	-	0.56 ± 0.16	0.94 ± 0.24	-	(20)

^*^Alfa Dominant females on follicular phase.

^**^Beta female on follicular phase.

Uterine variables: craniocaudal diameter (CCD) and the dorsoventral diameter (DVD) were measured by sagittal scan and the transversal diameter (TD) at the transversal scan.

**Table 3:** Mean and standard deviation of the ovary measurements in neotropical primates by ultrasound examination.

**Species**	**Length (cm)**	**Heigh (cm)**	**Width (cm)**	**Ovary Volume (cm^3^)**	**References**
*Aotus azarai infulatus*					
Right ovary	0.96 ± 0.11	0.55 ± 0.09	0.75 ± 0.12	0.21 ± 0.06	(5)
	0.99 ± 0.11	0.61 ± 0.08	0.79 ± 0.11	0.26 ± 0.0	
Left ovary	0.90 ± 0.12	0.90 ± 0.12	0.69 ± 0.10	0.19 ± 0.07	
	0.94 ± 0.11	0.94 ± 0.11	0.75 ± 0.11	0.23 ± 0.07	
Right ovary	-	-	-	0.32 ± 0.05	(6)
Left ovary	-	-	-	0.38 ± 0.09	
*Sapajus paella*	1.34 ± 0.02	0.82 ± 0.01	0.77 ± 0.01	0.45 ± 0.02	(10)
	1.22 ± 0.08	0.83 ± 0.05	0.91 ± 0.11	0.520 ± 0.01	(36)
*S. xanthosternos* and S. robustus^*^					(26)
Dominant female Right ovary^*^	0.91 ± 0.22	0.83 ± 0.11	0.69 ± 0.19	0.17 ± 0.07	
Dominant female Left ovary^*^	0.88 ± 0.26	0.72 ± 0.18	0.59 ± 0.15	0.15 ± 0.05	
Subordinate female Right ovary^*^	0.77 ± 0.20	0.67 ± 0.14	0.57 ± 0.16	0.17 ± 0.07	
Subordinate female Left ovary^*^	0.84 ± 0.37	0.69 ± 0.13	0.49 ± 0.15	0.15 ± 0.05	
*Saguinus ursulus*	0.69 ± 0.14	0.32 ± 0.06	0.53 ± 0.09	0.06 ± 0.02	(19)

^*^Follicular phase.

**Table 4:** Initial and final week of gestation according to fetal echo-biometric parameters (GSD, gestational sac diameters; CRL, crown-rump length; BPD, biparietal diameter; OFD, occipito-frontal diameter; HC, head circumference; AC, abdominal circumference; FL, femur length) and organogenesis measured by ultrasonography examination in eight species of neotropical primates.

	***Sapajus* sp. *(Cebus apella)***	***Saimiri bolivensis bolivensis***	***Callithrix jacchus***			***Saguinus fuscicollis***	***Aotus nancymaae***	***Aotus azarai infulatus***	***Leontoptecus* sp**.	***Sapajus apella***
**References**	**(** **23** **)**	**(** **21** **)**	**(** **38** **)**	**(** **12** **)**	**(** **13** **)**	**(** **20** **)**	**(** **9** **)**	**(** **39** **)**	**(** **27** **)**	**(** **25** **)**
**Parameters**	**Initial and final week of gestation**									
**GSD**	2.14–10.71	**-**	-	-	-	3–8		3–6	**-**	**-**
Latero-lateral longitudinal	**-**	-	**-**	**-**	**-**	**-**	**-**	**-**	-	3–8
Latero-lateral transversal	**-**	-	**-**	**-**	**-**	**-**	**-**	**-**	-	3–10
**CRL**	**-**	-	**-**	4.71–12.85	4.28–20.43	**–**	**–**	5–6	–	6–12
**BPD**	4.43–23.43	13.5–16.5	11.43–20.71	10–20.28	11.43–20.43	10–21	5.7–18.57	8–19	8–18	7–22
**OFD**	**-**	-	**-**	**-**	**-**	**-**	**-**	8–19	-	7–22
**HC**	**-**	-	**-**	**-**		**-**	**-**	8–19	-	7–22
**AC**	**-**	-	**-**	**-**	**-**	**-**	**-**	8–19	-	7–22
**FL**	10.71–23.43	-	**-**	**-**	**-**	**-**	**-**	10–19	-	10–22
**Fetal heart**	**-**	-	-	-	-	8–21	5.71–18.5	**-**	-	6–22
**Stomach**	**-**	-	**-**	**-**	**-**	**-**	**-**	**-**	-	6–22
**Urinary bladder**	**-**	-	**-**	**-**	**-**	**-**	**-**	**-**	-	7–22
**Lung**	**-**	-	**-**	**-**	**-**	**-**	**-**	**-**	-	≈11–22
**Liver**	**-**	-	**-**	14-?	**-**	**-**	**-**	**-**	-	≈11–22
**Kidney**	**-**	-	**-**	14-?	**-**	**-**	**-**	**-**	-	≈11–22
**Bowel**	**-**	-	**-**	**-**	**-**	**-**	**-**	**-**	-	≈11–22
**Fetal sex identification**	**-**	-	-	-	-	-	-	**-**	-	≈11–22

? Refers there is no information regarding the last week of pregnancy.

Considering the New World or Neotropical primates, 42.2% are listed as threatened, according to the International Union for Conservation of Nature (40). A small group of Neotropical (e.g., *Aotus* sp., *Callithrix jacchus, Saguinus* sp., *Saimiri* sp., and *Sapajus* sp.) primates is an important model for biomedical research because of its applicability. Only for these species are there studies on reproductive physiology and biotechnology that employ gynecological and obstetric ultrasonography as a tool (Tables 2–4). For instance, this paper discusses the state-of-the-art of gynecological and obstetric ultrasonography in the Neotropical primates, species that are models for biomedical research, and some recent studies on species targets for conservation strategies for wild animal populations.

### 1.1 Technical considerations

The transducer types and frequencies are important in gynecological and obstetric examinations by ultrasound. They should be selected based on the anatomy of the region to be examined and the body weight and size of the animal (Table 1). Linear probes have been the most used because they are the ones that ultrasound manufacturers make available with the highest frequency (4–9, 19, 35, 41). The linear probes require a relatively large contact area on the body surface, which is challenging for small neotropical primates. In contrast, micro convex probes may be better because it requires a smaller contact area. However, the available micro convex probes are generally low frequency, making their use in small neotropical primates unsuitable. Thus, a high-frequency micro convex probe may be a good choice for females of neotropical primate species that reach a maximum of 3 kg (10). The technological development of ultrasound equipment and the use of multi-high-frequency probes can be important in facilitating uterine and ovarian evaluations because they allow good-resolution images that can be associated with modern image analysis techniques. Thus, higher frequencies allow for a better definition but less penetration. Studies on Neotropical primates indicated multi-high-frequency probes from 5 to 18 MHz as the most utilized (Table 1). It is sometimes necessary to conduct ultrasound examinations using more than one type of transductor (11, 18, 20, 25, 38).

Although ultrasound examination does not cause pain, the decision to use chemical restraints can be made because some females show extremely stressed behavior during capture. A chemical restraint protocol can be implemented to avoid escape and excessive stress and to protect the animals and the investigator/assistant. However, using anesthetic drugs is associated with abortions (4). Therefore, small primates (e.g., marmosets and owls, and squirrel monkeys) can be trained to be handled and scanned without sedation (5, 38). The establishment of methods for conditioning females for ultrasound examination may provide a solution to this problem. Voluntary cooperation reduces the need for physical restraint and/or anesthesia and, therefore, the risks associated with these events (42). For instance, in owl monkeys (*Aotus azarai infulatus*), the method of offering fruits served as positive reinforcement, leading the animals to associate ultrasound examination with something pleasurable and stimulating (5). Reinforcement using food or liquids is called primary reinforcement, owing to its immediate biological consequences. This was compared with the decrease in the number of stress indicators observed in the experiment.

## 2 Gynecological ultrasonography (anatomical considerations)

### 2.1 Uterine examination

**Figure 2:**
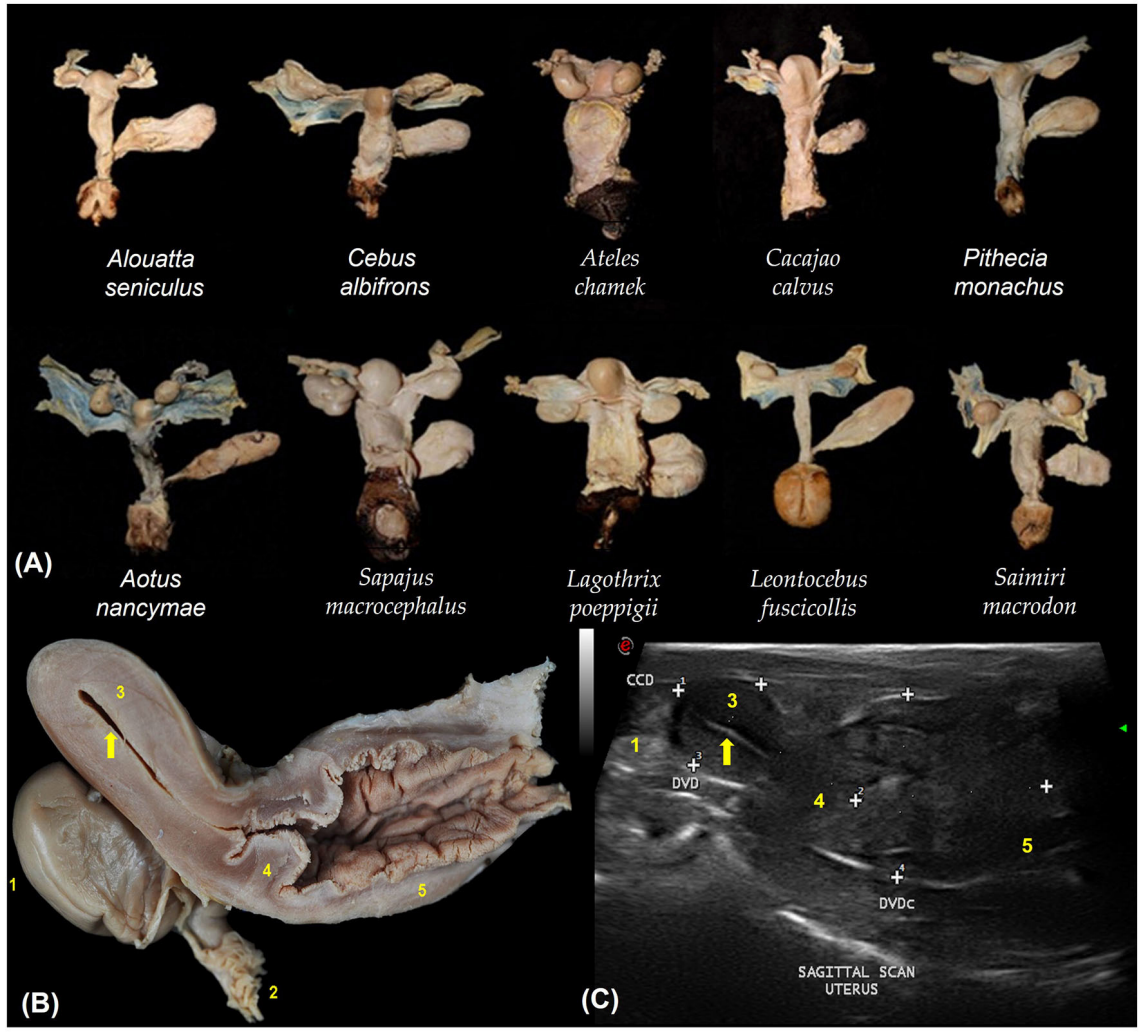
**(A)** Dorsal view of the urogenital organs of 10 non-pregnant females of different primate species. **(B)** Sagittal section of the urogenital organs in a wooly monkey female (*Lagothrix poeppigii*). Adapted from Mayor et al. (43). **(C)** Shows the uterine variables by sagittal scan ultrasound in no pregnant squirrel monkey (*Saimiri sciureus*). The cranio-caudal diameter (CCD), dorso-ventral diameter (DVD) and the cervix and vaginal dorso-ventral diameter (DVDc). Adapted from Pereira da Silva et al. (44). 1. Ovary; 2. Fallopian tube infundibulum (not visible by ultrasound); 3. Uterine body; 4. Cervix; 5. Vagina; Endometrium (arrows).

The urogenital tracts of primates are similar across species (Figure 2A). They have a simplex uterus (without uterine horns) with a pear-shaped appearance (globular fundus and more elongated base near the cervix; Figure 2B). The two fallopian tubes open into the fundus of the uterus and are not visible on ultrasound. The cervix is characterized by a thick wall that isolates the uterus from the vagina (Figure 2C). Uterine evaluation has always been a significant concern in gynecology and obstetrics because it is responsible for the maintenance of pregnancy and may be affected by various diseases that reduce fertility or cause severe damage to female health (22). Although ultrasonography is non-invasive, the need for sedation in some species and the high cost of equipment often hinder its routine utilization in some facilities (5).

The Neotropical primate uterus is located at the midline of the body between the urinary bladder and rectum (4, 10, 45). A moderately full urinary bladder is an important landmark for the ultrasonographic identification of the uterus (10), because the uterus is dorsal to the urinary bladder (18). The uterus of Neotropical primates is pear-shaped, and it is possible to distinguish the perimetrium, myometrium, and endometrium (5, 6, 10, 19, 24, 45). Considering that the uterus is a tubular organ, an experienced sonographer can visualize the uterine contents in specific pathological or physiological situations. The uterine parameters evaluated included the outline, shape, echogenic texture, position, linear dimensions, and volume (6, 10, 45).

As a cavitary organ, B-mode ultrasound examination of the uterus distinguishes its content from the presence of liquids, free masses, or masses adhered to the endometrium. The uterine layers are ultrasonographically distinct, and high-frequency probes (8–18 MHz) preferentially recognize the myometrium, endometrium, and lumen (19). The endometrium of a healthy, non-pregnant uterus appears as a single line, hyperechoic compared with the myometrium, cutting medially through the uterus. The endometrium can be analyzed for thickness, echotexture, and the presence of focal or disseminated abnormalities, masses, or cystic lesions. The myometrium can be evaluated for its echotexture and the presence of masses or cystic lesions. Changes in the uterine contour can be detected using B-mode ultrasonography, and it is necessary to report these changes (45).

**Figure 3:**
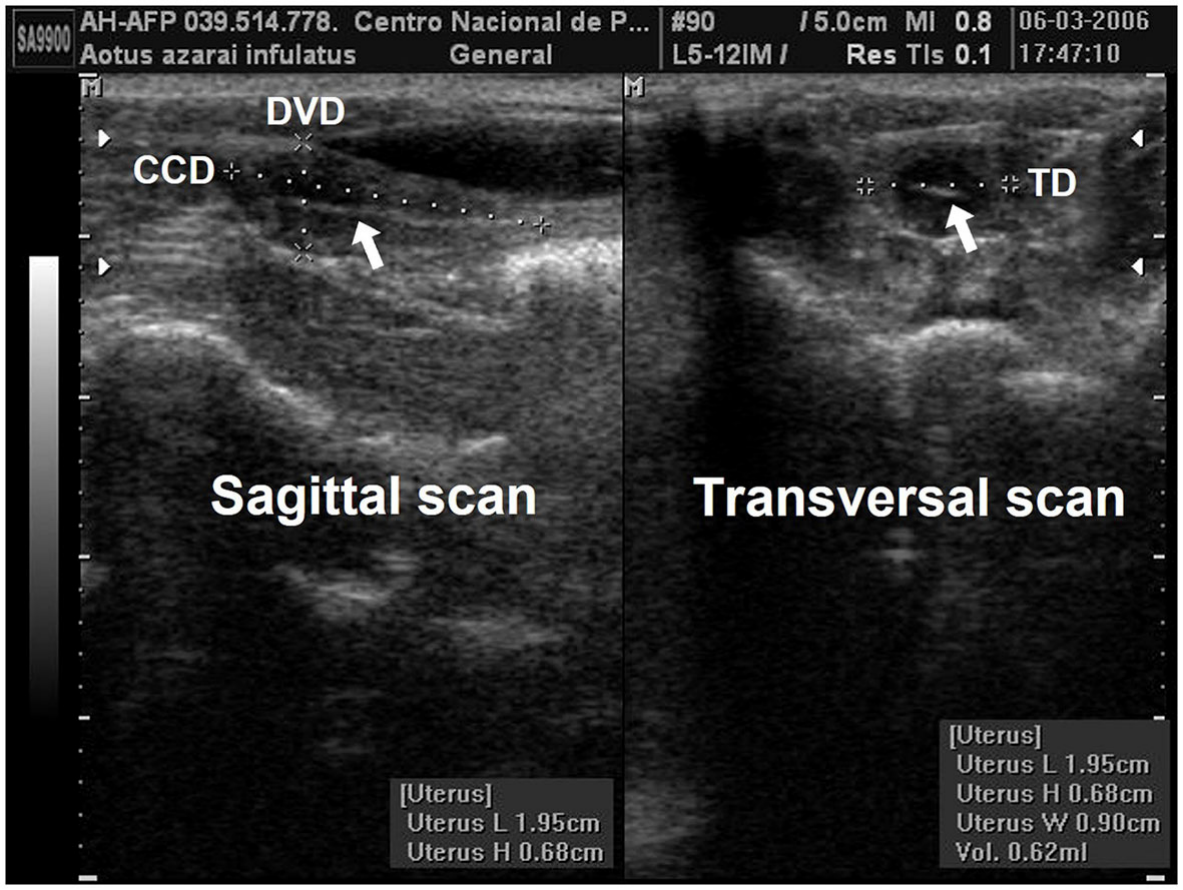
Uterine and ovarian examination in an adult female owl monkey. **(A)** Uterine variables: craniocaudal diameter (CCD) and the dorsoventral diameter (DVD) were measured by the sagittal scan. **(B)** The transversal diameter (TD) by the transversal scan. The hyperechogenic line in the central uterine region indicates the endometrium internal surfaces (arrow).

Linear uterine measurements and volumes were the principal parameters evaluated (Table 2). The variables representing the uterine length [craniocaudal diameter (CCD)] and height [dorsoventral diameter (DVD)] on sagittal scans and width [transverse diameter (TD)] on the transverse scan of the uterus (Figures 3A, B) are used to obtain the uterine volume (UV) calculus by the formula for an ellipsoid [p/6 (length × width × height)] (5, 10). Differences in the UV among nulliparous, primiparous, and multiparous females have been described in *Aotus azarai infulatus* (45). The UV of owl monkeys is proportional to the number of parturitions (4, 46). Regarding the linear measurements of the uterus of neotropical primates measured by B-mode ultrasonography, only three genera of primates (*Aotus* sp., *Sapajus* sp., and *Saguinus*) have been described in the literature (Table 2), which show the necessity of studies in other species.

Neoplasms that lead to changes in the size and shape of the uterus can be detected by assessing the uterine dimensions and volumes (22). Another important application for monitoring the uterine dimensions is the early diagnosis of embryonic death because the uterine dimensions and volume cease to increase or even start to regress when there is death and embryonic resorption (4).

### 2.2 Ovarian examination

**Figure 4:**
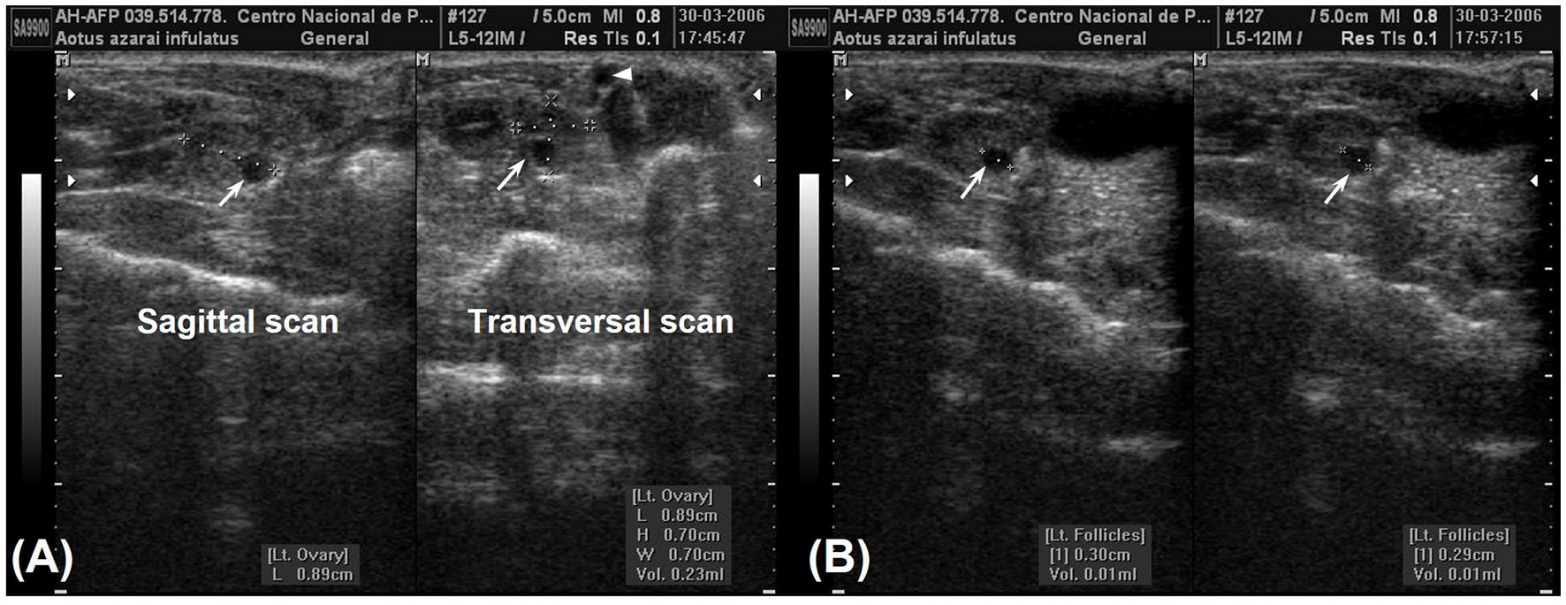
Ovaries ellipsoid with regular and well-defined contours in owl monkeys. **(A)** Shows the measurement of the left ovary in the sagittal and transverse sections, where a follicle (thin arrows) and iliac artery (arrowhead) are evident. **(B)** Sagittal section measurements of the internal diameter of the ovarian follicle [29–30 cm) located in the left ovary of the same female. Follicle showed through the echogenicity contrast between the follicular fluid (anechoic) and the ovarian tissue (hypoechoic).

The ovaries are oval or ellipsoid in shape in the NHPs (Figures 4A, B). The literature describes the Neotropical monkey ovaries as large structures, considering the size of the individuals, with an oval shape and dimensions according to the physiological stage (e.g., presence or absence of follicles and/or CL). Ovarian ultrasonography offers opportunities to study structural changes within the ovary during the sexual cycle. Linear ovarian measurements follow the uterus (Table 3), and the ellipsoid formula is commonly employed to determine the ovarian volume (5, 10, 45).

The follicular phase affects the volume of the ovary that houses the dominant follicle. Changes in ovarian volume are primarily attributed to follicular development (10). In contrast, Ortiz et al. (24) reported no statistically significant difference between the dominant and non-dominant ovaries on ultrasonography. These differences between the studies may be attributed to the methodology used to determine the ovarian volume. Some studies use only one linear dimension to measure ovarian volume (24). In contrast, all three linear dimensions (length, height, and width) to calculate the ovarian volume are more sensitive to predicting changes in the ovarian volume (10). Changes in the ovarian volume during the reproductive phases suggest that the females are not senescent and are an essential parameter for evaluating female reproductive health (5, 6, 10, 19, 26, 36).

The ability to recognize ovarian events using ultrasonography has evolved with the establishment of preovulatory antral follicles. Ovulation is a critical component for developing reproductive biotechnology, such as artificial insemination, hormonal ovary stimulation, and ovum pickup (10). The diagnosis of ovulation is challenging, and serial ultrasonographic examinations are necessary. The dominant follicle was a round or slightly ovoid anechoic structure with a well-delineated border within the ovary. The CL appeared to be a more echodense structure with an irregular border. The ultrasonographic findings indicated that ovulation occurred as follows: (1) disappearance or sudden decrease in the size of the preovulatory follicle, (2) increased echodensity, and (3) irregularity of the follicular walls (10). The estimated day of ovulation was defined as the day of maximum follicular size, followed by the day of evidence of follicular rupture (10, 47). In *A. azarai infulatus*, follicles >0.4 cm in diameter that disappeared before the subsequent examination and did not reappear with a larger or developing follicle were considered ovulatory (6). The dominant follicle was readily apparent 6 days before ovulation in *Sapajus apella* (10, 24). The follicular diameters increased from 4.3 to 10.1 mm, and the volumes ranged from 0.042 to 0.55 mL (or cm^3^) during the follicular phase (10).

B-mode ultrasonography is suitable for follicular growth monitoring, even in small Neotropical primates, such as *C. jacchus*. Ovulating follicles were already visible 4 days (−4) before ovulation. The mean diameter of these follicles increased from 2.0 ± 0.2 mm on day −4 to 3.0 ± 0.5 mm on day −1 (11). Follicular growth monitoring after hormonal stimulation is another important application of B-mode ultrasound, mainly in seasonal species, such as *Saimiri* sp., representing an additional difficulty in reproduction programs (8).

Ultrasound-guided ovum pickup has rarely been reported in Neotropical primates. The transabdominal ultrasound approach has been described for ovum pickup in *Saimiri boliviensis boliviensis* (41) and *A. azarai infulatus* (7). Surgical oocyte pickup was developed in *S. apella* after B-mode ultrasound monitoring of preovulatory follicular growth. Ovarian cortex biopsies were performed using the same procedure to study oocyte maturation, preantral follicular cryopreservation, and culture. In both studies, ultrasonography was essential for monitoring follicular growth and functional ovarian recovery after surgery (10, 36).

The application of Doppler ultrasonography in reproductive health and physiology studies of female Neotropical primates is of great importance in the female choice to participate in reproductive programs (10, 36). However, hemodynamic studies of the female reproductive organs in Neotropical primates are scarce. The iliac and uterine arteries have been studied in *A. azarai infulatus* (6) and *S. apella* (10, 25), respectively. The UOL artery has been studied in *S. apella* owing to its importance in controlling ovulation (10, 48). These studies revealed important hemodynamic events during the periovulatory period. Specifically, in *S. apella*, the blood supply increases in the UOL ipsilateral to the ovary, lodging the preovulatory dominant follicle near the ovulation period (10).

## 3 Obstetric ultrasound

The use of pregnant primates and their fetuses in scientific research has remained constant over the last few years. However, one of the major drawbacks of NHPs is that they can be extremely difficult and even dangerous to handle. Manual and/or chemical restraint is necessary and desirable to protect both the investigator and animal. According to Unwin et al. (49), manual restraint should not be considered for any NHP species weighing >5 kg or for any species not used to be handled. In addition, some parameters, such as physiological status (pregnant or not), size, and age, should be considered when deciding whether to anesthetize. For instance, during pregnancy, it is necessary to be careful with manual and/or chemical restraints, as they can cause pregnancy loss. In owl monkeys, anesthetic drugs and capture methods used to monitor pregnancy using ultrasound act as chemical and environmental factors that result in abortions (4). Thus, some small primates (marmosets and owl and squirrel monkeys) can be trained to be handled and, hence, scanned without sedation. Training them to cooperate voluntarily using positive reinforcement training techniques is one means of significantly reducing the adverse impact of physical stress and anesthetic drug. These actions are important for monitoring and understanding the primate gestational physiology (39).

Reproductive applications of ultrasound in NHP breeding colonies can provide an efficient method for pregnancy detection, fetal monitoring during gestation, and routine assessment of breeding females (50). Evaluating the efficacy of ART is crucial, and ultrasound is the method of choice for female cycle monitoring, early pregnancy diagnosis, and conceptus development study (11). Ultrasonography has been used to study gestation in *C. jacchus* (11–13), *Saguinus fuscicollis* (20), *Aotus azarae infulatus* (4, 39), *Aotus nancymaae* (9), *Leontopithecus rosalia, Leontopithecus chrysomelas, Leontopithecus chrysopygus* (27), *Saimiri* sp. (21), *Sapajus* sp. (*Cebus apella*) (23), and *S. apella* (25). With increased studies on NHP pregnancy failures (37) and experimental Zika virus studies (51), adequately describing normal and abnormal conceptus growth and fetal development is crucial.

**Figure 5:**
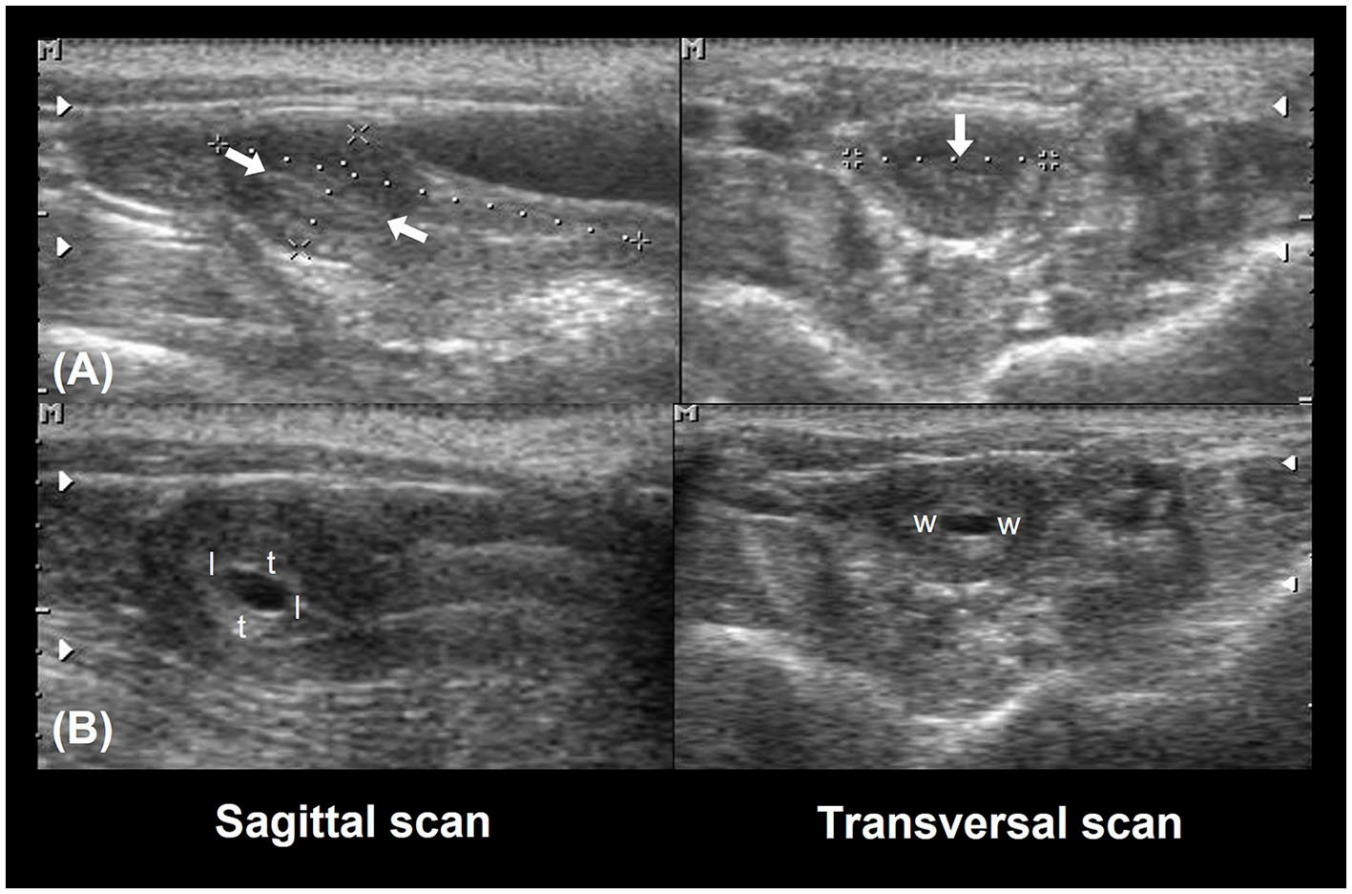
**(A)** The second week of gestation of owl monkey, showing the increase in the uterine variables (CCD, DVD, TD, and UV) and the endometrial thickness (ET, arrow), which are considered early gestational signs. **(B)** The fundus implantation of the GS in the 3rd week of gestation of owl monkeys, showing length (L) and height (T) by sagittal scan and the width (W) by the transverse scan of gestational sac diameters.

Serial ultrasonographic sessions were conducted to identify early ultrasonographic signs of gestation (EUSG) and developing ultrasonographic signs of gestation (DUSG). EUSG was considered if the endometrial thickening and UV increased (4) (Figure 5A). Evidence of the gestational sac (GS) and visualization of the embryonic bottom were considered DUSG (4) (Figure 5B). The diameters of the GS (CCD, DVD, and TD) or latero-lateral longitudinal and latero-lateral transversal diameters are the mean parameters for monitoring conceptus growth before fetal morphogenesis (4, 20, 23, 25, 39) as showed in Table 4.

**Figure 6:**
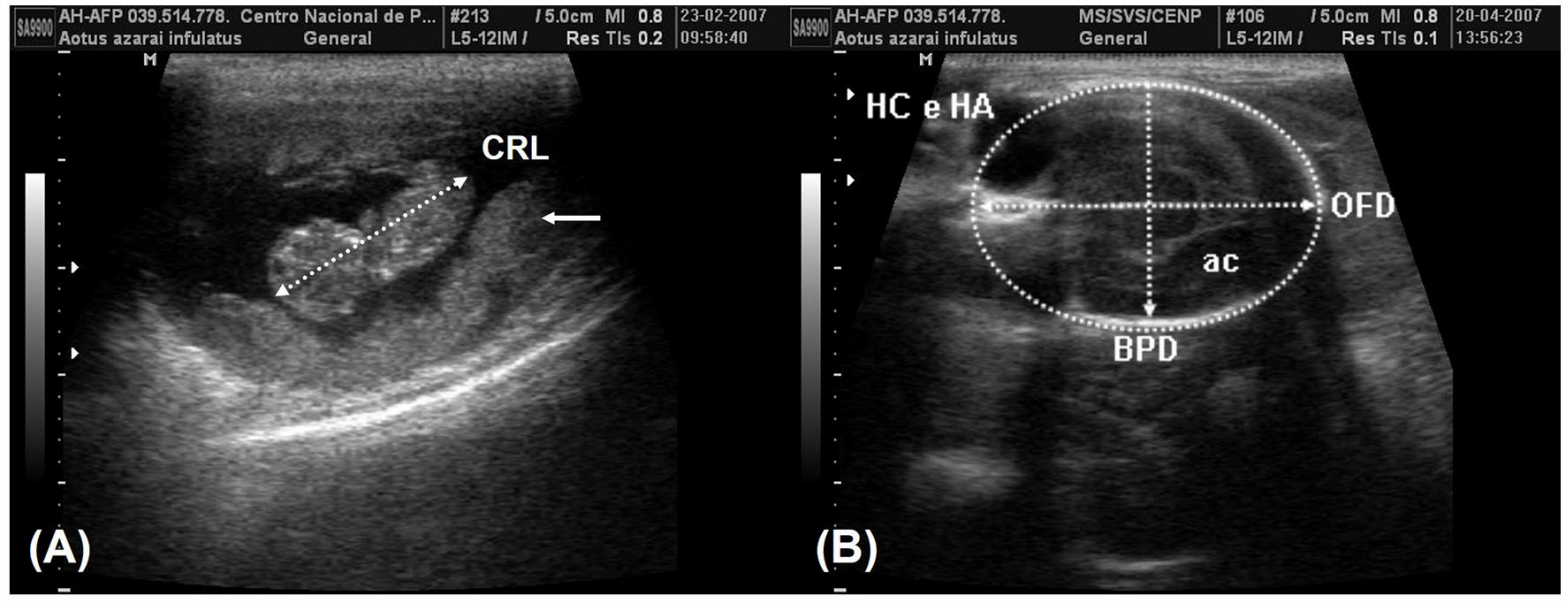
**(A)** The crown-rump length (CRL) measurement in the 8th week of gestation and the placenta (arrows). **(B)** The plane of the thalamic exam in the 16th week of gestation shows the ambient cistern (ac) in the posterior section of the head. The biparietal diameter (BPD) was obtained by positioning the probe from the external surface of the proximal cranial table to the distal internal surface. The occipitofrontal diameter (OFD) was obtained by measuring the external margins of the fetal cranium perpendicular to the BPD. The head circumference and head area (HC and HA) were obtained in the same examination by tracing around the external border of the hyperechogenic outline in the fetal cranium border.

**Figure 7:**
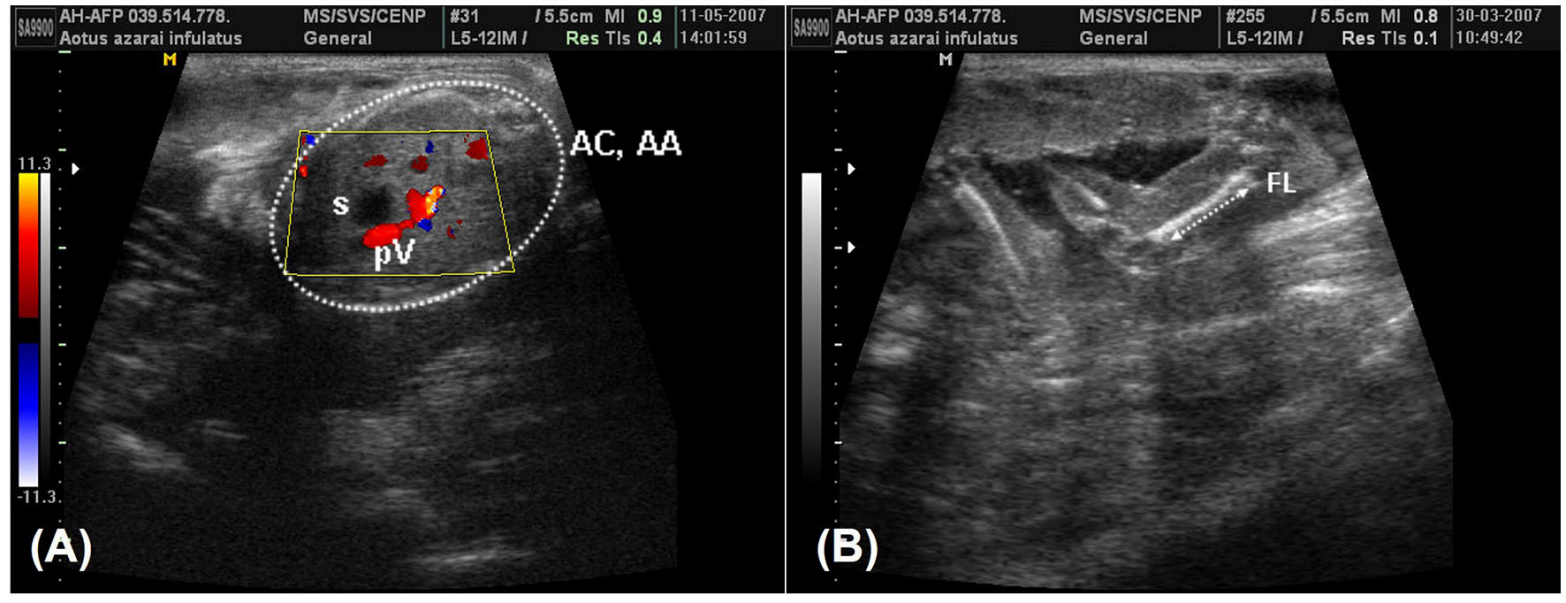
**(A)** A color-Doppler image during the 19th week of owl monkey gestation that was used to facilitate identifying the umbilical portion of the portal vein (pV) in the liver and the fetal stomach (s). The abdominal circumference and the abdominal area (AC and AA) were measured in the fetal abdômen transverse scan perpendicular to the vertebral axis (arrow). **(B)** The femur length (FL) in the 13th week of gestation was measured from the proximal and distal extremities, measuring only the femoral diaphysis.

According to Monteiro et al. (39) and Miranda et al. (25), fetal development parameters can be useful for monitoring pregnancy, such as following the crown–rump length (CRL) on the longitudinal section of the fetus, which is obtained from the top of the cranium to the base of the tail (Figure 6A). The biparietal diameter (BPD) and occipitofrontal diameter (OFD) were measured in the thalamic plane, determined by the ambient cistern appearance at the rear of the head (Figure 6B). The BPD was measured by placing calipers from the external surface of the proximal cranial table to the distal internal surface. The OFD was obtained by measuring the distance from one external fetal cranial margin to the other margin perpendicular to the BPD. The head circumference and head area were measured by delineating the fetal cranium's hyperechoic outline in the same scan used for the BPD and OFD. The abdominal circumference and abdominal area were measured by placing the probe around the external portion of the hyperechogenic border of the fetal abdomen (Figure 7A). A transverse scan perpendicular to the vertebral axis was obtained from the fetal abdomen. This scan visualizes the stomach and the umbilical portion of the portal vein in the liver. Color Doppler mode can be used to easily identify the umbilical portion of the portal vein (Figure 7A). The femoral length (FL) was measured from the distal and proximal diaphysis extremities (Figure 7B).

In a study of prenatal development in capuchin monkeys, ultrasonography was used (23). The authors observed the embryonic development and the beginning of fetal development. In six animals, pregnancy was diagnosed between 15 and 27 days of gestation. The GS was visible at a mean of 23.7 ± 2.8days. Embryonic development was evaluated using the largest SG and CRL. Fetal development was evaluated using the CRL, BPD, thoracic height, and FL. The authors concluded that ultrasonography is a useful diagnostic method for determining and assessing the gestational age in this species. The capuchin monkey follows the general embryonic development plan of mammals, and its fetal development is chronologically similar to that observed in other primates (*Macaca fascicularis, Macaca* mulatta, and *Papio anubis*), with similar gestational periods (52, 53).

Kuederling and Heistermann (20) studied the gestation of *S. fuscicollis* (142–150 days), comparatively, by ultrasound and plasmatic progesterone dosage, and verified that ultrasonographic examination was suitable for earlier for pregnancy diagnosis. After conception, the endometrial surfaces separated and formed a lumen, which was detected between 16 and 18 days of gestation (17.2 ± 1.0 days), when uterine cavity had a small circular hypoechoic structure in the middle of the endometrial line, a safe indication of gestational sac and pregnancy. The amniotic sac was visualized between the fourth and 5th weeks, at ~32–52 days of gestation. One week before the estimated time of conception, low progesterone levels were detected, with mean values of 4.7 ± 1.6 ng/mL. A marked postconception increase was observed, resulting in a mean level of 107.1 ± 46.0 ng/mL around day 24 of gestation. After that, the progesterone levels decreased to 71.6 ± 30.3 ng/mL and remained at this plateau until ~2 months before delivery, when a second significant increase occurred. Peaks of 278.5 ± 67.3 ng/mL were found 1 month before delivery, declining to levels close to preconception levels (7.4 ± 7.8 ng/mL) within a week after delivery.

The mean gestational and fetal echo-biometry parameters evaluated in neotropical primates are shown in Table 4. The serial evaluation gestational sac was described only for *Sapajus* sp. (23, 25), *Saguinus fuscicollis* (20), and *Aotus azarai* infulatus (39). In the same way, for the fetal echo-biometry, the biparietal diameter is the mean parameter for monitoring fetal development in neotropical primates. In *Callithrix jacchus*, the BPD was the best parameter to relate with the gestational age, showing that the diagnosis of a small BPD for gestational age results in fetal death 7 days after birth (13). However, other parameters important to diagnosing intrauterine growth restriction, such as AC and HC, are described just for *Sapajus* sp. and *Aotus* sp. It is important to notice that a small AC for gestational age may reflect a reduction in the size of the liver or other intra-abdominal organs, and HC reflects the head size and brain growth (54). Microcephaly, for example, is diagnosed when the HC is below the mean for gestational age (55). In neotropical primates, studies about HC related to gestational age still need to be completed. Recently, craniofacial malformation was diagnosed by B-mode ultrasound measurement of HC, OFD, and BPD in *Saimiri collinsi* experimentally infected with ZIKV in the second trimester of gestation (51).

Fetal echo-biometry ultrasound monitoring during the gestational period may be suitable for predicting the whelping time using regression analysis formulas (Table 5). Nonetheless, considering the lack of studies on fetal echobiometry in neotropical primates, the use of ultrasound to predict whelping time is limited to *Aotus nancymae* (9), *Leontoptecus* sp. (27), and *Sapajus* sp. (23, 25).

**Table 5:** Regression analyses regarding the relationship between gestational age (days of gestation or weeks before whelping—WBW) and gestational sac diameters (GSD), crown-rump length (CRL), biparietal diameter (BPD), occipito-frontal diameter (OFD), head circumference (HC), abdominal circumference (AC), femur length (FL) in three species of neotropical primates.

	***Aotus nancymae***	***Leontoptecus* sp**.	***Sapajus*** **sp**.	
**Parameters**	(9)	(27)	(23)	(25)
**GSD**	**-**	**-**	Day: 19.1064 + 12.6309 GS	
Latero-lateral longitudinal	-	-	-	11.46 ± 0.45 WBW
Latero-lateral transversal	-	-	-	9.52 ± 0.38 WBW
**CRL**	-	-		16.93 ± 0.93 WBW
**BPD**	Day: 12.11 + (43.66 × BPD) + [(BPD – 2.07)^2^ × 4.21]	−8.679+1.7633^*^Week	Day: 20.6935 + 31.8825 BPD	16.93 ± 0.93 WBW
**OFD**	-	-	-	5.84 ± 0.27 WBW
**HC**	-	-	-	16.68 ± 0.80 WBW
**AC**	-	-	-	13.46 ± 0.67 WBW
**FL**	-	-	Day: 47.8507 + 27.6 FL	3.62 ± 0.22 WBW

Fetal wellbeing is usually monitored based on the fetal heartbeat (23). Relationships between the fetal size, fetal wellbeing, and characteristics of blood flow in the uterine artery (UA) and umbilical artery evaluated by triplex Doppler showed suitability for evaluating fetal viability using 2D B-mode ultrasonography (25). Among the Doppler indices, the pulsatility index (PI) and resistivity index (RI) are commonly used for obstetric applications. As described for humans, in capuchin monkeys (*Sapajus* sp.), an early diastolic notch is ordinarily present in the UA wave flow until the second trimester of gestation. The early diastolic notch disappears concurrently with significant decreases in the RI and PI of the UA (25).

## 4 Final considerations

In summary, this review shows the importance of ultrasound for understanding reproductive physiology in neotropical primates. However, it also arises that studies on ultrasonography applied to non-human primate reproduction are almost restricted to species used in biomedical research. Even in the biomedical research primates species, there species, there is a lack of studies about ovulation predicted day, early diagnosis of gestation, echo-biometry parameters to determine gestational age, fetal vitality, and whelping time, as well as studies involving Doppler parameters for ovarian, uterine, and umbilical arteries. It is important to notice that all these aspects are essential for the development of reproductive programs related to the conservation of these non-human primates, and in biomedical research such as studies on the effect of the Zika virus on the fetal development of both human and non-human primates.

## Author contributions

SD, FM, and DL contributed to conception and design of the study and wrote the first draft of the manuscript. DL organized the database. FM wrote sections of the manuscript and contributed with figures. All authors contributed to manuscript revision, read, and approved the submitted version.

## References

[1] IUCN/SSC. Primates, Specielist Group. (2023). Available online at: http://www.primate-sg.org/who_ares_the_primates/ (accessed June 09, 2023).

[2] Groves CP. Order primates. In:Wilson DE Reeder DM, editors, Mammal *Species*. of the *World*: A *Taxonomic*. and *Geographic Reference*. 3rd ed. Baltimore, MD: *J.H.U. Press*. (2005). p. 111–84.

[3] Rylands AB Mittermeier RA Silva Jr JS. Neotropical primates: taxonomy and recently described species and subspecies. Int Zoo Yearb. (2012) 46:1–14. 10.1111/j.1748-1090.2011.00152.x

[4] Monteiro FOB Koivisto MB Vicente WRR Carvalho RA Whiteman CWW Castro PH. Uterine evaluation and gestation diagnosis in owl monkey (*Aotus azarai infulatus*) using the B mode ultrasound. J Med Primatol. (2006) 5:123–30. 10.1111/j.1600-0684.2006.00155.x 16764669

[5] Monteiro FOB Coutinho LN Pompeu ESS Castro PHG Maia CE Pereira WLA. Ovarian and uterine ultrasonography in *Aotus azarai infulatus*. Int J Primatol. (2009) 30:327–36. 10.1007/s10764-009-9346-1

[6] Coutinho LN de Brito MBS Monteiro FOB de Andrade RS da Conceição MEB Feliciano MR. Analysis of follicular events in owl monkeys (*Aotus azarai infulatus*) using B-mode and doppler ultrasound. Theriogenology. (2013) 80:99–103. 10.1016/j.theriogenology.2013.03.018 23602218

[7] Rech F Souto MP Oliveira JWM Silva SKSM Furtado PV Imbeloni AA. Ultrasonography-guided oocyte recovery in owl monkeys (Aotus azarai infulatus). J Med Primatol. (2021) 50:134–7. 10.1111/jmp.12509 33432651

[8] Schuler AM Westberry JM Scammell JG Abee CR Kuehl TJ Gordon JW. Ovarian stimulation of squirrel monkeys (*Saimiri boliviensis boliviensis*) using pregnant mare serum gonadotropin. Comp Med. (2006) 56:12–6. 16521854

[9] Schuler AM Brady AG Tustin GW Morris CG Abee CR. Measurement of fetal biparietal diameter in owl monkeys (*Aotus nancymaae*). J Am Assoc Lab Anim Sci. (2010) 49:560–3. 20858355 PMC2949423

[10] Domingues SF Caldas-Bussiere MC Martins ND Carvalho RA. Ultrasonographic imaging of the reproductive tract and surgical recovery of oocytes in *Cebus apella* (capuchin monkeys). Theriogenology. (2007) 68:1251–9. 10.1016/j.theriogenology.2007.08.023 17915305

[11] Nubbemeyer R Heistermann M Oerke A-K Hodges JK. Reproductive efficiency in the common marmoset (*Callithrix jacchus*): a longitudinal study from ovulation to birth monitored by ultrasonography. J Med Primatol. (1997) 26:139–46. 10.1111/j.1600-0684.1997.tb00045.x 9379480

[12] Jaquish CE Toal RL Tardif SD Carson RL. Use of ultrasound to monitor prenatal growth and development in the common marmoset (*Callithrix jacchus*). Am J Primatol. (1995) 36:259–75. 31924098 10.1002/ajp.1350360402

[13] Tardif SD Jaquish CE Toal RL Layne DG Power RA. Estimation of gestational ages in the common marmoset (*Callithrix jacchus*) from published prenatal growth curves. J Med Primatol. (1998) 27:28–32. 9606040 10.1111/j.1600-0684.1998.tb00065.x

[14] Windle CP Baker HF Ridley RM Oerke AK Martin RD. Unrearable litters and prenatal reduction of litter size in the common marmoset (*Cul1ithui.xjacchus*). J Med Primatol. (1999) 28:73–83. 10431697 10.1111/j.1600-0684.1999.tb00254.x

[15] Takahashi T Hanazawa K Inoue T Sato K Sedohara A Okahara J. Birth of healthy offspring following *ICSI in* in vitro matured common marmoset (Callithrix jacchus) *oocytes. PLoS ONE*. (2014) *9*:e95560. 10.1371/journal.pone.0095560 PMC3994092 24751978

[16] Hastings JM Morris KD Allan D Wilson H Millar RP Fraser HM. Contrast imaging ultrasound detects abnormalities in the marmoset ovary. Am J Primatol. (2012) 74:1088–96. 10.1002/ajp.22063 22890799

[17] Ishibashi H Motohashi HH Kumon M Yamamoto K Okada H Okada T. Ultrasound-guided non-surgical embryo collection in the common marmoset. Reprod Biol. (2013) 13:139–44. 10.1016/j.repbio.2013.02.002 23719119

[18] Grandez RR Rosales ER Merino VO Hermoza CG Muñoz KD. Descripción de las características ultrasonográficas de los órganos abdominales del machín blanco (*Cebus albifrons*). Rev Inv Vet Perú. (2021) 32:e2004. 10.15381/rivep.v32i2.20043

[19] Borges LB Pereira AKF Silva WB Monteiro FOB Coutinho LN. Abdominal ultrasound in *Saguinus ursulus*. J Med Primatol. (2020) 2020:1–8. 10.1111/jmp.12487 32881001

[20] Kuederling I Heistermann M. Ultrasonography and hormonal monitoring of pregnancy in the saddle back tamarin, *Saguinus fuscicollis*. J Med Primatol. (1997) 26:299–306. 10.1111/j.1600-0684.1997.tb00058.x 9438223

[21] Lögdberg B. Methods for timing of pregnancy and monitoring of fetal body and brain growth in squirrel monkeys. J. Med. Primatol. (1993) 22:374–379. 8138988

[22] Long CT Luong RH McKeon GP Albertelli MA. Uterine leiomyoma in a guyanese squirrel monkey (*Saimiri sciureus sciureus*). J Am Assoc Lab Anim Sci. (2010) 49:226–30. 20353700 PMC2846013

[23] Corradini P Recabarren M Serb-Ferrt M Parraguez VH. Study of prenatal growth in the capuchin monkey (*Cebus apella*) by ultrasound. J Med Primatol. (1998) 27:287–92. 10.1111/j.1600-0684.1998.tb00077.x 10203008

[24] Ortiz RE Ortiz AC Gajardo G Zepeda AJ Varraguez VH Ortiz ME. Cytologic, hormonal, and ultrasonographic correlates of the menstrual cycle of the new world monkey Cebus apella. Am J Primatol. (2005) 66:233–44. 10.1002/ajp.20141 16015660

[25] Miranda SA Leão DL Oliveira KG Sodré IS Domingues SFS. Gestational ultrasonography and Dopplerfluxometry in capuchin monkeys (*Sapajus apella*) zoometric. Theriogenology. (2018) 108:63–73. 10.1016/j.theriogenology.2017.11.023 29197294

[26] Pissinatti TA Ribas JAS Maróstica E Pissinatti A Ferreira AMR. Ultrasound monitoring of the uterus and ovaries of dominant and subordinate females of yellow-breasted capuchin (*Sapajus xanthosternos*) and robust tufted capuchin (*Sapajus robustus*) in captive colonies during the ovarian cycle and anestrus periods. Pesq Vet Bras. (2019) 39:989–96. 10.1590/1678-5150-PVB-6174

[27] Luz MS Vidal FD Burity CHF Bobány DM Pissinatti A. Ultrasonographic aspects of the Leontopithecus gestation (Lesson, 1840—Callitrichidae, Primates). J Med Primatol. (2017) 2017:1–5. 10.1111/jmp.12319 28972670

[28] Sheiner E Hackmon R Shoham-Vardi I Pombar X Hussey MJ Strassner HT. A comparison between acoustic output indices in 2D and 3D/4D ultrasound in obstetrics. Ultrasound Obstet Gynecol. (2007) 29:326–8. 10.1002/uog.3933 17265534

[29] Pooh RK Kazuo M Kurjak A Sen C Ebrashy A Adra A. 3D/4D sonography – any safety problem. J Perinatal Med. (2015) 44:125–9. 10.1515/jpm-2015-0225 26376219

[30] Miranda SA Domingues SFS. Conceptus ecobiometry and triplex Doppler ultrasonography of uterine and umbilical arteries for assessment of fetal viability in dogs. Theriogenology. (2010) 74:608–17. 10.1016/j.theriogenology.2010.03.008 20494430

[31] Barbosa CC Souza MB Freitas LA Silva TFP Domingues SFS Silva LDM. Assessment of uterine involution in bitches using B-mode and Doppler ultrasonography. Anim Reprod Sci. (2013) 139:121–6. 10.1016/j.anireprosci.2013.02.027 23602011

[32] Barbosa CC Souza MB Scalercio SRRA Silva TFD Domingues SFS Silva LDM. Ovarian and uterine periovulatory Doppler ultrasonography in bitches. Pesq Vet Bras. (2013) 33:1144–50. 10.1590/S0100-736X2013000900016

[33] Szatmári V Sótonyi P Voros K. Normal duplex Doppler waveforms of major abdominal blood vessels in dogs: a review. Vet Radiol Ultrasound. (2001) 42:93–107. 10.1111/j.1740-8261.2001.tb00911.x 11327368

[34] Hildebrandt TB Drews B Kurz J Hermes R Yang S Göritz F. Pregnancy monitoring in dogs and cats using 3D and 4D ultrasonography. Reprod Domest Anim. (2009) 44:125–8. 10.1111/j.1439-0531.2009.01429.x 19754550

[35] Schuler AM Parks VL Abee CR Scammell JG. Ultrasonographic monitoring of a spontaneous abortion in an owl monkey (*Aotus nancymaae*). J Am Assoc Lab Anim Sci. (2007) 46:74–6. 17645301

[36] Santana LN Brito AB Brito DC Lima JL Domingues SFS Santos RR. Adaptation of a trap door technique for the recovery of ovarian cortical biopsies from *Cebus apella* (capuchin monkey). Zygote. (2013) 21:158–61. 10.1017/S0967199411000724 22475413

[37] Tardif S Power M Layne D Smucny D Ziegler T. Energy restriction initiated at different gestational ages has varying effects on maternal weight gain and pregnancy outcome in common marmoset monkeys (*Callithrix jacchus*). Br J Nutr. (2004) 92:841–9. 10.1079/bjn20041269 15533274

[38] Oerke AK Einspanier A Hodges JK. Noninvasive monitoring of follicle development, ovulation, and corpus luteum formation in the marmoset monkey (*Callithrix jacchus*) by ultrasonography. Am J Primatol. (1996) 39:99–113. 10.1002/(SICI)1098-2345(1996)39 31918495

[39] Monteiro FOB Coutinho LN Silva GA Castro PHG Maia CE Silva KSM. Ultrasound evaluation of pregnancy in owl monkey (*Aotus azarai infulatus*). Anim Reprod. (2011) 8:40–6.16764669

[40] Mittermeier RA Reuter KE Rylands AB Jerusalinsky L Schwitzer C Strier KB. Primates in Peril: The World's 25 Most Endangered Primates 2022–2023. Washington, DC: IUCN SSC Primate Specialist Group, International Primatological Society, Rewild (2022).

[41] Schuler AM Westberry JM Parks VL Kuehl TJ Abee CR. Ultrasound-guided follicular aspiration in squirrel Monkeys. J Med Primatol. (2007) 36:113–7. 10.1111/j.1600-0684.2007.00219.x 17493142

[42] Laule GE Bloomsmith MA Schapiro SJ. The use of positive reinforcement training techniques to enhance the care, management, and welfare of primates in the laboratory. J Appl Anim Welf Sci. (2003) 6:163–73. 10.1207/S15327604JAWS0603_02 14612265

[43] Mayor P Plana L Silva C Pereira G Monteiro T Ozanan F. ATLAS DE ANATOMIA DE ESPÉCIES SILVESTRES AMAZÔNICAS Volume III (versão PORTUGUÊS)-Órgãos Urinários e Órgãos Genitais. Edufra, Belém (2022).

[44] Pereira da Silva G Souza Pereira TH de Felipe Lima AK Vicente WRR Kuehl TJ Ruiz JC. Female squirrel monkeys as models for research on women's pelvic floor disorders. Lab Anim. (2021) 55:499–508. 10.1177/00236772211032506 34323623 PMC12371763

[45] Monteiro FOB Coutinho LN Silva ASL Domingues SFS Miranda SA. Ultrassonografia do Sistema Reprodutor em Primatas N3o-humanos. In: U*ltrassonografia na Reprodução Animal. 1st ed*. MedVet (2013).

[46] Mauad-Filho F Beduschi AF Meschino RAG Mauad FM Casanova MS Ferreira AC. Avaliação ultra-sonográfica das variações do volume uterino. Rev Bras Ginecol Obstet. (2001) 23:175–9. 10.1590/S0100-72032001000300007

[47] Baerwald A Adams G Pierson R. A new model for ovarian follicular development during the human menstrual cycle. Fertil Steril. (2003) 80:116–22. 10.1016/s0015-0282(03)00544-2 12849812

[48] Nagle CA Digiano L Paul N Terlato M Quiroga S Mendizabal AF. Interovarian communication for the control of follicular growth and corpus luteum function in the Cebus monkey. Am J Primatol. (1994) 34:19–28. 31936986 10.1002/ajp.1350340106

[49] Unwin S Ancrenaz M Bailey W. Handling, anaesthesia, health evaluation and biological sampling. In Setchell J, Curtis D, editors, Field and Laboratory Methods in Primatology: A Practical Guide. Cambridge: Cambridge University Press (2011). p. 147–68.

[50] Tarantal AF. Ultrasound imaging in rhesus (*Macaca mulatta*.) and long-tailed (*Macaca fascicularis*.) macaques: reproductive and research applications. In: Coote SW, e*d*itor, T*he Laboratory Primate. San Diego, CA: Elsevier*. (2005). p. 317–52.

[51] Imbeloni AA Alcantara BN Coutinho LN Scalercio SRA Carneiro LA Oliveira KG. Prenatal disorders and congenital Zika syndrome in squirrel monkeys. Sci Rep. (2021) 11:2698. 10.1038/s41598-021-82028-3 33514824 PMC7846595

[52] Tarantal AF Hendrickx AG. Prenatal growth in the cynomolgus and rhesus macaque (*Macaca fascicularis* and *Macaca mulatta*): a comparison by ultrassonography. Am J Primatol. (1988) 15:309–23. 10.1002/ajp.1350150405 31968883

[53] Herring JM Fortman JD Anderson RJ Bennett T. Ultrasonic determination of fetal parameters in Baboons (Papio anubis). Lab Anim Sci. (1991) 41:602–5. 1667206

[54] Roberts AB Mitchell JM McCowan LM Barker S. Ultrasonographic measurement of liver length in the small-for-gestational-age fetus. Am J Obstet Gynecol. (1999) 180:634–8. 10.1016/S0002-9378(99)70266-8 10076140

[55] Melamed N Yogev Y Danon D Mashiach R Meizner I Ben-Haroush A. Sonographic estimation of fetal head circumference: how accurate are we? Ultrasound Obstet Gynecol. (2011) 37:65–71. 10.1002/uog.7760 20661958

